# Determining bacteriophage endopeptidase activity using either fluorophore-quencher labeled peptides combined with liquid chromatography-mass spectrometry (LC-MS) or Förster resonance energy transfer (FRET) assays

**DOI:** 10.1371/journal.pone.0173919

**Published:** 2017-03-15

**Authors:** Rolf Lood, Henrik Molina, Vincent A. Fischetti

**Affiliations:** 1 Laboratory of Bacterial Pathogenesis and Immunology, The Rockefeller University, 1230 York Avenue, New York, NY, United States of America; 2 Department of Clinical Sciences Lund, Division of Infection Medicine, Lund University, Lund, Sweden; 3 Proteomics Resource Center, The Rockefeller University, 1230 York Avenue, New York, NY, United States of America; Centro Nacional de Biotecnologia, SPAIN

## Abstract

The necessity of identifying novel methods to combat infections caused by antibiotic resistant bacteria is increasing each year. Recent advancements in the development of peptidoglycan hydrolases (*e*.*g*. lysins) from bacterial viruses (bacteriophages) have revealed the efficiency of this class of enzymes in treating serious bacterial infections. Though promising results have been obtained regarding the lethal action of lysin on bacterial pathogens both *in vitro* and *in vivo*, an often-overlooked factor in these studies is precisely identifying their peptidoglycan cleavage site. This knowledge would be useful for following the activity of the enzyme during development, without the need for whole-organism lytic assays. However, more importantly, it would enable the selection of lysins with different cleavage activities that would act synergistically for enhanced efficacy. Here, we have developed two new methods to accurately identify the cleavage site of lysins using liquid chromatography mass spectrometry (LC-MS) on peptidoglycan-like fluorophore-quencher modified synthetic peptides, as well as determining the enzymatic action and kinetics of the enzymes on modified peptides in a Förster resonance energy transfer (FRET) assay. These methods should facilitate progress within the lysin field, accelerating the development of therapeutic lysins to combat antibiotic resistant bacterial infections.

## Introduction

One of the current threats humanity faces is the rapid increase of antibiotic resistance seen amongst pathogenic microorganisms [1]. This, in combination with the marked reduction in the development of novel antibiotics [2], has encouraged the scientific community to investigate novel alternatives for treating antibiotic resistant infections. One such alternative is the use of bacteriophage lysins [3]. Bacteriophages are viruses that specifically infect bacteria, propagate within their host, and subsequently escape the bacteria by the production of a peptidoglycan hydrolase resulting in hypotonic lysis and the release of progeny phages [4]. Peptidoglycan hydrolases can have four distinct activities, cleaving the peptidoglycan between the sugar moieties (Endo-β-*N*-acetylglucosaminidase or *N*-acetylmuramidase), between the sugar and the stem peptide moieties (*N*-acetylmuramoyl-L-alanine amidase) or between any of the amino acids in the stem peptide or cross bridge (endopeptidases). The efficiency of several lysins has been formally demonstrated *in vivo*, rescuing animals from lethal bacterial infections [5–8]. However, for most lysins, there is a lack of information regarding what bond(s) they hydrolyze in the peptidoglycan. This knowledge gap may be attributed to a lack of easily accessible methods to identify and characterize the cleavage sites. In recent years, we and others have developed several new methods to help facilitate this research [9], based on the addition of bulky reactive substrates after cleavage of the peptidoglycan [10] or by changes in isotopic patterns [5]; all analyzed by mass spectrometry. Even though these new methods have facilitated the characterization of lysins, they still require specific knowledge and advanced mass spectrometry analyses [5,10], and are thus partly limiting our advances in this research field.

When studying bacteriophage endolysins, a common method to evaluate the lysin’s activity is to test its capacity to degrade the peptidoglycan, by lysing and killing the bacterium. This is usually studied by measuring ability of the lysin to reduce the optical density of a bacterial suspension, followed by plating the surviving bacteria and calculating the killing efficiency. While important, this assay does not specifically identify the lysin’s ability to act on the peptidoglycan, and suffers from issues of reproducibility. Bacteria are highly surface decorated, with proteins, lipids and carbohydrates, masking access to their peptidoglycan. The extent of decoration differs from strain to strain, and growth phase [11]. Thus, while an optical density reduction assay evaluates the ability of the lysin to lyse specific bacterial strains at a certain growth phase, it does not specifically evaluate the cleavage activity of the lysin on the peptidoglycan.

To overcome this problem and establish a more reproducible method to study lysin activity, we developed two methods based on fluorophore-quencher pair labeled peptides, adapted for analysis on either liquid chromatography mass spectrometry (LC-MS) or Förster resonance energy transfer (FRET) assays. In FRET assays, we take advantage of the ability of the quencher (dabcyl) to act on the fluorophore (EDANS) in close proximity at either end of a peptide. Once the distance between the two molecules is vastly increased (*e*.*g*. by proteolytic activity) the quencher is no longer able to inhibit the fluorophore, resulting in a fluorescent signal. This has been commonly used to study several proteases [12–15], and recently for a bacterial autolysin with amidase activity [9], but has yet to be evaluated for the study of phage endopeptidases. While this method is useful to determine if a cleavage has occurred within a FRET-modified peptide, it does not reveal precisely where the cleavage has occurred. However, we found that the addition of the quencher dabcyl and the fluorophore EDANS at either end of the peptide increased the hydrophobicity of their attached peptides after cleavage, making them better suited for C18-based reversed phase chromatography to identify the exact cleavage site.

As a proof of concept, we studied three distinct peptidoglycan hydrolases: the *Staphylococcus simulans* bacteriocin lysostaphin [16], the chimeric *Staphylococcus aureus* phage lysin ClyS [17], and the *Streptococcus suis* phage lysin PlySs2 [7], all with demonstrated activity on the Gram-positive pathogen *S*. *aureus*. Lysostaphin has already been shown to be a glycyl-glycine endopeptidase [18], ClyS (containing the Twort phage lysin CHAP domain) has not yet been formally characterized, but was suggested to be a D-alanyl-glycine endopeptidase based on homology to phage lysin phi11 [19], and the cleavage site for PlySs2 has not been characterized. Further, to verify that the obtained results are dependent on the specific peptide substrate, we included a *Streptococcus pyogenes peptidoglycan* peptide and studied the effect of the *S*. *pyogenes* lysin PlyPy and the *Streptococcus suis* lysin PlyPy on this peptide.

Here we demonstrate the efficacy and ease of use of a high-throughput method to study the cleavage site(s) of peptidoglycan hydrolases using LC-MS on cleavage products from peptidoglycan-like synthetic peptides digested with the four different peptidoglycan hydrolases, as well as FRET assays for the study of lysin kinetics and enzymatic characteristics.

## Materials and methods

### Proteins

Lysostaphin was purchased from Sigma-Aldrich (St. Louis, MO, USA), ClyS was purified as described elsewhere [17], and PlySs2 and PlyPy were purified as described in Gilmer et al [7] and Lood et al [5], respectively. All enzymes were analyzed for their activity on their respective bacterial cells to ensure their activity.

### Peptides

All peptides were synthesized by GenScript (Piscataway, New Jersey, NJ, USA). Briefly, peptides were synthesized on Cl-resin with a Solid-Phase Peptide Synthesis protocol using Fmoc to protect the amino acids, and piperidine for deprotection. The coupling was enabled by diisopropylcarbodiimide (DIC) and 1-hydroxybenzotriazole (HOBT). Peptides were purified through reverse phase chromatography (RP-HPLC). In order to visualize peptides, the software SpecViewer (Advanced Chemistry Development) was used.

### FRET assays

Peptides analyzed in FRET assays had N-terminal Dabcyl and C-terminal EDANS attached to the peptide backbone. Assays were performed in a quartz 96-well plate (Molecular Devices, Sunnyvale, CA, USA) in 20 mM Tris-HCl pH8.0 supplemented with 1 mM CaCl_2_. Fluorescence was measured on a SpectraMax M5 96-well plate reader (Molecular Devices) at 340/490 nm, shaking and measuring every other minute for 3 hours at 37°C.

### Mass spectrometry

Peptides (4 μg) were incubated with ClyS, PlySs2, PlyPy, or lysostaphin (10 μg) overnight at 37°C in 20 mM Tris-HCl pH 8.0, supplemented with 1 mM CaCl_2_. Enzymes were removed before analysis through ultrafiltration on 10 kDa MWCO filters. The samples were injected on to and separated by a C18 column (Acclaim 120: 120Å, 2.1x150 mm, Dionex) connected to a mass spectrometer (OrbitrapXL, ThermoFisher) operated in positive ESI mode (ESI potential: 4.0 kV). In both MS-only and MS/MS (CID) only, ions were measured at a resolution of 60,000@m/z 400. Peptides were eluted at 200 μl/min using a 14-minute linear gradient increasing from 0% buffer B/100% buffer A (A: 0.1% formic acid, B: 0.1% formic acid in acetonitrile) to 40% buffer B/60% buffer A. In between each analysis the column was cleaned by ramping the solvent to 90% buffer B/10% buffer A in 1 minute followed by washing for 3 minutes at this composition. The column was hereafter conditioned for 5 minutes at 100% buffer A. All used solvents were of HPLC grade. MS ion traces for all potential fragments were calculated and extracted using Skyline [20]. Both N- and C-terminal fragments were included in the analysis. All samples were analyzed in technical triplicates.

## Results and discussion

### Generation of synthetic peptides

**Fig 1:**
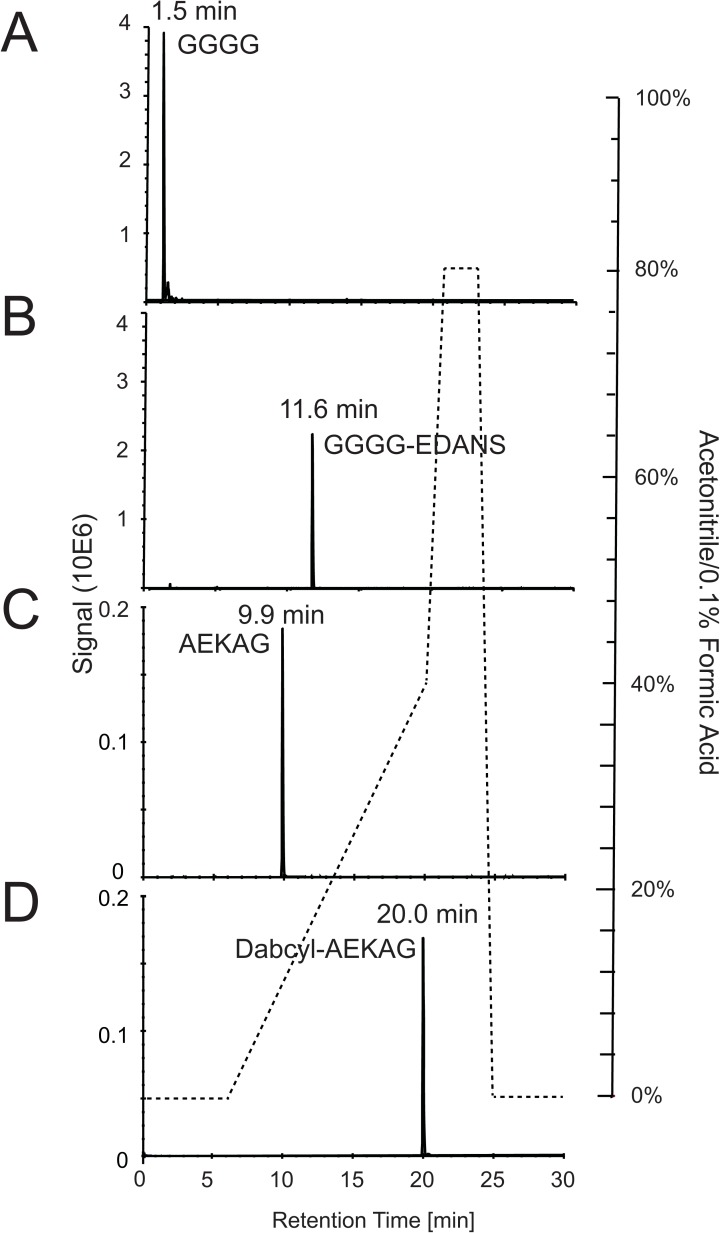
Addition of FRET-molecules to the synthetic peptides increases hydrophobicity. Native (A, C) and FRET-labeled (B, D) peptides were digested with lysostaphin, and separated by reversed phase C18 chromatography. Samples were loaded onto the column using 0.1% formic acid for 6 minutes, where after the solvent composition was changed to 40% buffer B (acetonitrile, 0.1% formic acid) and 60% buffer A (0.1% formic acid) over 14-minutes, depicted in the figure (see right y-axis for scale). Selected extracted ion trace representing N- and C-terminal fragments for the primary hydrolyzed peptides are shown.

A peptide corresponding to the *S*. *aureus* stem peptide with its attached pentaglycine cross-bridge was synthesized (AEKAGGGGG). To use the peptide for FRET analyses, the FRET pair dabcyl and EDANS was attached N- and C-terminally, respectively. These modifications were found to simplify downstream LC-MS analysis due to their added hydrophobicity allowing more efficient analysis by C18 reversed phase chromatography. An unmodified “native” peptide was also analyzed by LC-MS. When comparing the lysostaphin digestion fragments of the native synthetic peptide with the digested FRET-labeled peptides, a dramatic increase in the retention time (>10 minutes) of the FRET-labeled peptide was observed (Fig 1), demonstrating their increased hydrophobicity. In this instance, the peptide used only represents a portion of the peptidoglycan, which can only identify endopeptidase activities. However, it has earlier been demonstrated that N-terminal modifications of such a peptide may allow for the identification of amidases and glycosidases [21]. Furthermore, since the binding domain has a high affinity for its wall receptor, its influence on the catalytic domain comes from its ability to efficiently drive the catalytic domain to its cell wall substrate. Thus, the effectiveness, not the bond specificity, of the catalytic domain is influenced by the presence of the binding domain [22].

### Cleavage patterns of different endopeptidases based on LC-MS

**Fig 2:**
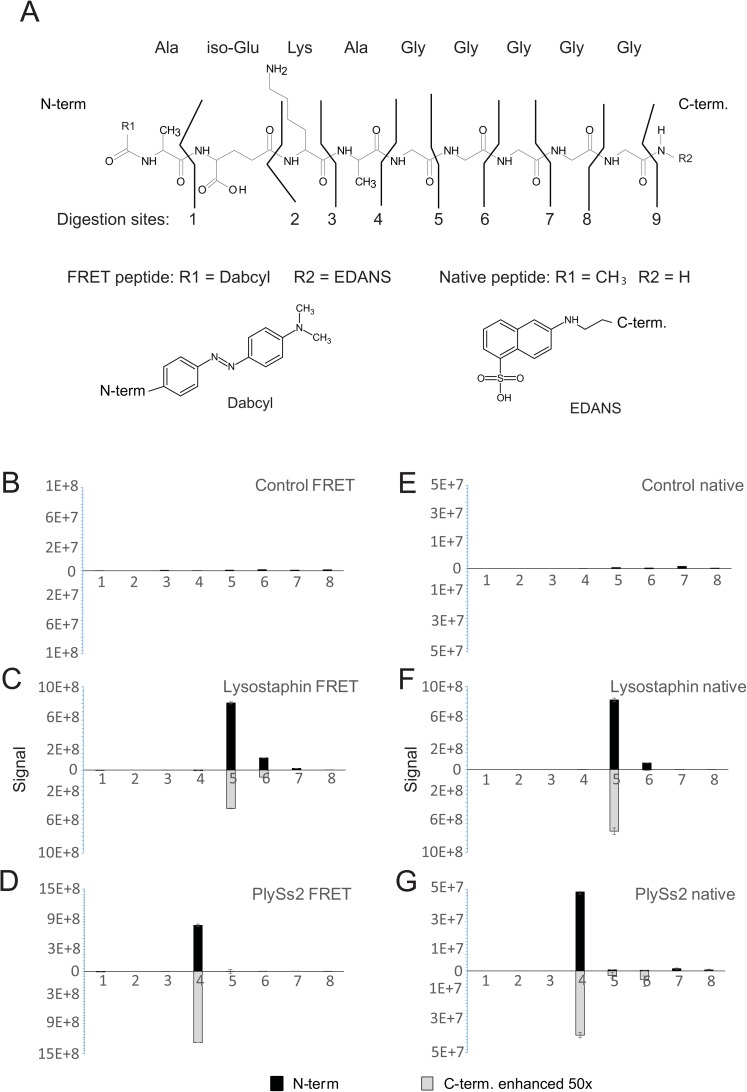
Cleavage patterns of different staphylococcal endopeptidases using FRET-labeled and native peptides. Peptidoglycan-like peptides (A) were incubated overnight with buffer only (B, E), lysostaphin (C, F), or PlySs2 (D, G). Both FRET-labeled (B-D), and native peptides (E-G) were used. Generated fragments were detected using LC-MS and analyzed using Skyline. Panels B through G: Measured signals are shown on the y-axis. N-terminal fragments are shown as positive values (black bars) while fragments corresponding to the C-termini are shown with light grey bars. Because the C-terminal signals were found to be very low, the values were multiplied by 50 for visualization purposes. Standard deviations for the replicated measurements are shown. The x-axis shows the origin of the signal with respect to the peptidoglycan-like peptide.

To determine the cleavage site(s) of the staphylococcal endopeptidases, FRET-labeled peptides were incubated overnight with three different enzymes: with known (lysostaphin), suggested (ClyS), and unknown (PlySs2) activities (Fig 2A–2D). To facilitate the mass spectrometry analysis, enzymes were removed from the reaction by ultrafiltration before analysis. We found that lysostaphin was able to degrade most of the target peptide, generating several minor fragments, demonstrating its ability to act on several bonds in the glycine cross-bridge, with a preference for the first glycyl-glycine bond attached to the D-alanine. However, diminishing activity on the second and third bond was also observed (Fig 2C), as demonstrated earlier [18]. Unlike lysostaphin, PlySs2 demonstrated a digestion pattern indicative of a D-Ala-Gly endopeptidase activity, with the main ion identified corresponding to dabcyl-AEKA (Fig 2D), cleaving the bond between the stem peptide and the cross-bridge. However, peptides digested with ClyS repeatedly failed to generate ion signals significantly higher than background suggesting that this peptide was not efficiently hydrolyzed by ClyS. For all samples, both N-terminal and C-terminal hydrolytic fragments of the peptide from individual samples were measured. This allowed for a more confident assessment that the signals observed were accurate, and not falsely identified peaks.

Though the data indicates a D-Ala-Gly endopeptidase activity for PlySs2, it is unlikely to be the only enzymatic activity employed by this particular lysin. PlySs2 is able to kill *S*. *aureus* both *in vitro* and *in vivo* [7], however, it also has broad activity against several *Streptococcus* and *Listeria* species which lack this particular cross-bridge sequence.

**Fig 3:**
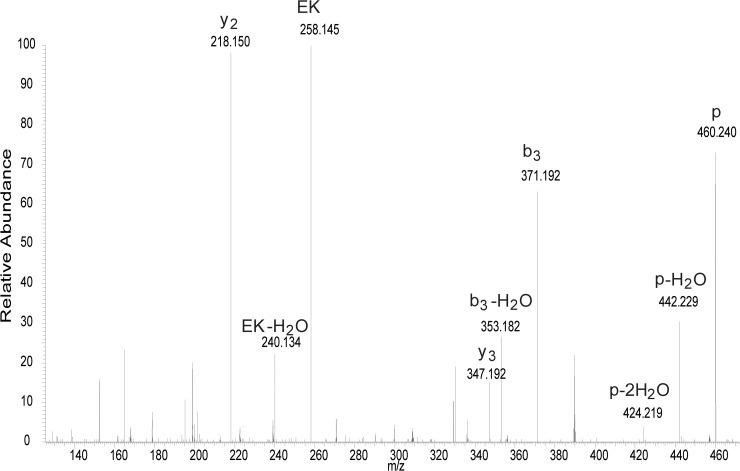
PlyPy can hydrolyze a *Streptococcus pyogenes* like peptidoglycan. A non-labeled N-terminal acetylated peptide resembling the *S*. *pyogenes* peptidoglycan within the stem peptide and crossbridge (AEKAAA) was incubated with the *S*. *pyogenes* lysin PlyPy, and hydrolysis of the peptide was measured through mass spectrometry (MS). After incubation, an ion appeared at m/z 460.2404 which was matched as acetyl-AEKA (0.6 ppm). This interpretation was confirmed by tandem MS experiments. In the shown tandem MS, the precursor ion (p) as well as N-terminal (y) and C-terminal (b) fragment ions are marked. Fragment ions are matched with mass accuracies better than 2 ppm.

To demonstrate that the method was not specific for *S*. *aureus* peptidoglycan-like peptides, we synthesized a *Streptococcus pyogenes* peptidoglycan-like peptide which also included the stem peptide and cross bridge amino acids (AEKAAA). Reactivity of this peptide with the *S*. *pyogenes* specific lysin PlyPy resulted in a cleavage between the D-Ala and L-Ala (Fig 3), demonstrating a D-alanyl-L-alanine endopeptidase activity in accordance with recent data [5]. However, even though the broad-spectrum lysin PlySs2 is able to act upon the *S*. *pyogenes* peptidoglycan, we could not detect any hydrolysis of the AEKAAA synthetic peptide. Other CHAP domain enzymes have been found to have additional enzymatic activities within their single catalytic domain, being both endopeptidase and amidase [21]. Since the synthetic peptides used in this study do not allow for the measurement of an amidase or glycosidase, we cannot exclude the possibility that the broad spectrum cleavage seen for PlySs2 is due to its putative amidase or glycosidase activity. Neither can we exclude that binding to the glycan chain of the peptidoglycan is necessary for PlySs2 activity on this peptide. Further experiments will be needed to investigate this.

To verify that the presence of the added bulky FRET molecules attached to the peptides did not interfere with the lysin’s enzymatic activity, or in other ways negatively influenced the assay, we repeated the experiment using the staphylococcal peptides without any N- or C-terminal additions. While the data generated using this peptide was in perfect agreement with the FRET-labeled peptide (Fig 2E–2G), the measured signals for the enzymatically-generated fragments were 50–100 times lower, and thus more difficult to detect by ESI-MS. However, the N-terminal parts of both the native and the FRET-labeled peptides were consistently easier to detect. This was expected, since dabcyl contains a secondary amine in addition to the N-terminally located lysine residue in the peptide, which can stabilize protons, resulting in an increased hydrophobicity. In contrast, the C-termini of both native and FRET-modified peptides only contain amino acids without any primary amines (*e*.*g*. glycines), as well as the FRET molecule EDANS, lacking any proton acceptor at neutral pH. Therefore, we consistently isolated double positively charged (2+) ions as the highest signal for the N-terminal fragments. We did not, however, investigate if negative ESI would impact the efficiency of detecting C-terminal fragments.

### Using the FRET assay for lysin cleavage and enzyme kinetics

As mentioned earlier, one of the more common ways to study the lytic effects of phage endolysins is to measure the reduction in optical density (OD_600_) of whole bacterial cells after hypotonic lysis. While this data is important to judge the ability of a lysin to physically lyse a bacterium, it does not necessarily correlate with the lysin’s ability to cleave a peptidoglycan bond. Furthermore, the reproducibility of this particular experiment is partly limited, since bacteria grown to different OD_600_ will: i) express different surface molecules, ii) have a wide array of cell wall anchored proteins, and iii) vary in the extent of peptidoglycan crosslinking [23], which will affect the lytic assay.

**Fig 4:**
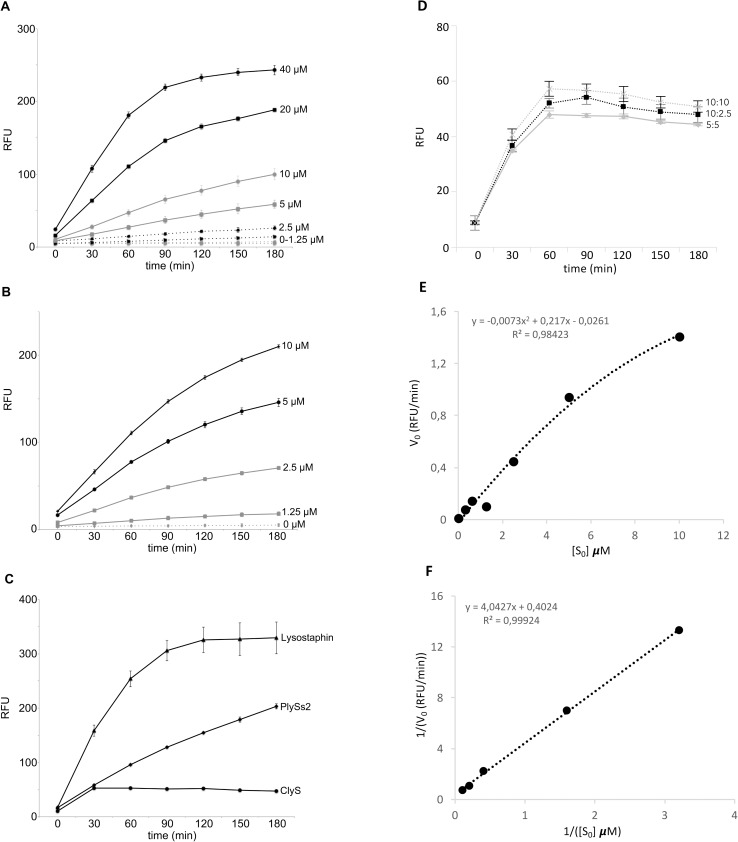
Förster Resonance Energy Transfer assays for peptidoglycan hydrolases. FRET assays were performed with the synthetic peptide Dabcyl-Ala-Glu-Lys-Ala-Gly_5_-EDANS in 20 mM Tris pH 8.0 supplemented with 2 mM CaCl_2_, and measured at 340/490 nm. A) The effect of varying concentration of PlySs2 (0–40 μM) in the presence of 5 μM peptide. B) The effect of varying concentration of peptide (0–10 μM) in the presence of 5 μM PlySs2. C) The kinetics of lysostaphin, ClyS and PlySs2 (5 μM) in the presence of 5 μM peptide. D) The kinetics of ClyS under a different ClyS:peptide ratio. E) The V_0_ of PlySs2 at different concentrations of peptide (substrate) was calculated based on the data points from Fig 4B, and plotted in a V_0_/[S_0_] graph, with a two degree polynomial function used to fit the data points. F) A Lineweaver-Burk plot of 1/V_0_ vs 1/[S_0_], with one outlier removed for clarity. The graph was used for calculating the k_cat_ and K_m_. All experiments were conducted in triplicates, at least three times, and representative figures are shown.

Rather than using whole peptidoglycan as a template, we synthesized peptide fragments as described above with an attached FRET pair at the termini (dabcyl-EDANS) and directly measured the increase in fluorescence after enzymatic cleavage. While this assay can be used to determine the cleavage activity for peptidoglycan hydrolases without the need for incorporating mass spectrometry analysis, only cleavage of the target substrate is revealed and not the precise cleavage site of the enzyme. However, the method is useful for studying lysin kinetics where varying the amount of peptide or enzyme increases the fluorescent signal in a dose dependent manner (Fig 4A and 4B). Such assays would be useful for measuring enhanced catalytic effects through increased substrate recognition from a protein engineering standpoint. Furthermore, the assay allows for an efficient comparison of different enzymes under identical conditions. Using 5 μM peptide and 5 μM enzyme, lysostaphin reached saturation after 3 hours, while the PlySs2-generated signal was still increasing (Fig 4C). However, ClyS consistently reached a plateau around 50 RFU (relative fluorescence units). An increase in peptide or ClyS concentration did not significantly affect this reaction (Fig 4D). The reduced ability of ClyS to digest the peptides may thus partly explain our lack of measurable ions (above background) in the LC-MS approach when peptides were treated with ClyS. Whether this relates to its chimeric nature, its inability to efficiently cleave this particular peptide, or the necessity of an intact native peptidoglycan as a substrate remains to be elucidated. Due to partly insoluble peptide particles, inter-experimental comparison may be partly limited. This limitation was substrate/enzyme specific, and not an inherited issue with the method described herein since we did not experience any such variation with the streptococcus peptide, and intra-experimental measurements had very little variance. However, prudence is needed in the choice of peptide buffer, to enable both a complete solubility of the peptide, while simultaneously not affecting the enzymatic activity.

Finally, using the generated data-set for PlySs2, we were able to calculate the speed by which fluorescence was generated per minute, and analyzed this in two different graphs (Fig 4E and 4F). Using a Lineweaver Burk plot, we calculated the K_m_ to 10 μM, and k_cat_ to 2.5 RFU/min. The K_m_ value is close to that reported for the endopeptidase lysostaphin (70 μM) [24], but significantly higher than the glycosidase lysozyme (16.7 nM) [25], indicating that the class of enzymatic activity (e.g. glycosidase or protease) may affect the K_m_ value.

## Conclusions

During recent years, research regarding phage lysins and their potential as a novel antibacterial therapy has been widely explored. However, the complete molecular characterization of the lysins themselves has been hampered due to the lack of easily accessible methods to study their cleavage site in the peptidoglycan and enzyme kinetics. Using FRET-modified peptidoglycan peptides, we demonstrated methods to easily identify the bonds cleaved by phage endopeptidases in the bacterial cell wall and measure the kinetics of this cleavage activity. This should help advance the bacteriophage lysin field and be a valuable tool for the continued study of these important enzymes.
